# A Culturally Tailored mHealth App (VCheck) to Support Breast Self-Examination Among Thai Women: Development and Formative Evaluation Study

**DOI:** 10.2196/92230

**Published:** 2026-08-12

**Authors:** Patamaporn Molee, Jitima Kositsukjaroen, Nattaporn Somjit

**Affiliations:** 1 Department of Radiological Technology, Faculty of Medicine Vajira Hospital, Navamindradhiraj University, 681 Samsen Road, Dusit, Bangkok, 10300, Thailand, 66 814467779; 2 Vajira Center of Excellence In Cancer Care, Faculty of Medicine Vajira Hospital, Navamindradhiraj University, Bangkok, Thailand; 3 Department of Radiology, Ratchaphiphat Hospital, Bangkok, Thailand

**Keywords:** breast self-examination, mobile health, smartphone, usability testing, Thailand

## Abstract

**Background:**

Breast cancer is a leading cause of mortality among Thai women. Breast self-examination (BSE) is a low-cost practice that promotes breast health awareness and familiarity with breast changes; however, adherence and correct technique remain suboptimal, partly due to limited guidance and reminder systems. Mobile health (mHealth) interventions could support BSE practice.

**Objective:**

This study aimed to develop VCheck, a culturally tailored mHealth app for Thai women, and conduct a formative evaluation of its usability and feasibility.

**Methods:**

A user-centered design framework guided the development of VCheck using the Thunkable cross-platform environment. The app included educational multimedia content, step-by-step BSE guidance, and a smart reminder system. A formative pilot usability study was conducted among 55 female volunteers aged 20 to 50 years over a 2-month period. Usability was assessed using the System Usability Scale. Feasibility and interface design were evaluated using a multidimensional satisfaction questionnaire, and qualitative feedback was collected.

**Results:**

The mean System Usability Scale score was 85.4 (SD 6.8), indicating excellent usability. Participants reported high overall satisfaction (mean 4.46, SD 0.68), including with feasibility (mean 4.48, SD 0.64) and interface design (mean 4.44, SD 0.72). Qualitative feedback suggested that the app was easy to navigate and culturally appropriate.

**Conclusions:**

In this formative evaluation, VCheck demonstrated high usability and user acceptance as a culturally tailored mHealth tool for breast health education and BSE awareness among Thai women. The findings support further refinement and larger-scale evaluation of behavioral and implementation outcomes.

## Introduction

Breast cancer remains the most commonly diagnosed malignancy among women worldwide and in Thailand, posing a substantial public health challenge. In 2022, approximately 2.3 million new cases and 666,103 deaths were reported worldwide, underscoring the persistent burden of breast cancer on women’s health [1]. In Thailand, the National Health Security Office reported 22,158 new cases of breast cancer in 2020, representing 22.8% of all newly diagnosed cancers among women [2]. Established risk factors, including age and genetic predisposition, are largely nonmodifiable. Moreover, early-stage breast cancer frequently remains asymptomatic, contributing to delayed diagnosis, presentation at advanced stages, and poorer clinical outcomes [3,4].

Women aged over 40 years, those with a family history of breast or ovarian cancer, and individuals with previous benign or malignant breast lesions are considered at higher risk [5,6]. The Thai Department of Health recommends that women initiate regular breast self-examination (BSE) from the age of 20 years [7]. BSE enables women to become familiar with the normal characteristics of their breasts and recognize changes that warrant professional evaluation [8]. Although primary prevention remains limited, timely clinical evaluation of breast changes is associated with improved survival outcomes. Current breast health surveillance approaches include BSE, clinical breast examination (CBE), and mammography, each playing different roles in breast health promotion and referral pathways [9]. While meta-analyses indicate that BSE alone does not significantly reduce breast cancer mortality, regular BSE practice has been associated with earlier tumor detection and a lower likelihood of advanced disease at diagnosis [10]. In low- and middle-income countries, where access to mammography is constrained, international guidelines continue to support BSE and CBE as pragmatic, low-cost strategies for promoting breast health awareness; however, their primary value in this context lies in increasing women’s familiarity with breast changes and facilitating timely referral for clinical evaluation rather than serving as stand-alone screening tools [9,11,12].

Despite national screening initiatives, adherence to monthly BSE among Thai women remains suboptimal [13]. At the same time, digital technologies are increasingly integrated into health promotion and preventive care, with mobile health (mHealth) apps offering scalable and accessible platforms for health education and behavioral interventions [14]. Thailand’s Ministry of Public Health has established a national eHealth and digital health strategy aimed at strengthening health care delivery through information and communications technologies, including mobile apps and integrated health information systems [15]. The World Health Organization has likewise identified digital health as a strategic priority in Thailand’s Country Cooperation Strategy, emphasizing the role of digital platforms in improving health care accessibility and service delivery [16]. These policy frameworks underscore the potential of mHealth interventions to support preventive care and health promotion services in Thailand. Previous studies have shown that mobile apps can improve breast cancer awareness, enhance confidence in performing BSE, and increase user engagement and satisfaction [17-19]. Nevertheless, many existing apps lack culturally localized content, reminder functions, and usability optimization for Thai users, which may hinder sustained engagement and adherence [14,20-22]. Cultural beliefs, privacy concerns, and variations in health literacy may further influence breast cancer screening behaviors among Thai women.

Existing mHealth tools such as BrAware, SmartBreast, and BSEApp primarily focus on information dissemination and provide limited localization or behavioral reinforcement mechanisms. To address these gaps, the VCheck app was developed using a user-centered design (UCD) approach incorporating Thai-language educational content, animated BSE demonstrations, and an integrated reminder system to enhance usability and sustain engagement. However, few mHealth solutions have been systematically culturally tailored and usability tested specifically for Thai women, and evidence regarding feasibility within local preventive health contexts remains limited.

mHealth refers to the use of mobile and wireless technologies to support health services, health education, and health-related behavior change [23]. Within preventive care, mHealth apps can provide accessible educational content, personalized reminders, self-monitoring tools, and interactive guidance. Self-care refers to the ability of individuals to maintain health, prevent disease, and manage health-related behaviors through informed decision-making and routine practices. In this study, VCheck was conceptualized as a self-care support tool for breast health awareness and regular BSE practice rather than as a diagnostic or screening replacement [24].

Therefore, this formative study aimed to develop and conduct a feasibility and usability evaluation of the VCheck mHealth app, a culturally tailored and user-centered mHealth prototype designed to support Thai women in performing regular BSE.

## Methods

### Study Design and Participants

This study used a formative research design consisting of three phases: (1) app design and content development, (2) prototype development, and (3) formative usability and feasibility evaluation among potential end users.

Women aged 20 to 50 years who met the inclusion criteria were eligible to participate. A convenience sample of 55 participants was recruited, which is consistent with recommended sample sizes for formative mHealth usability studies [25]. A minimum sample size of 48 was estimated using the formula for determination of sample size for estimating a finite population mean (*n* = *NZ*^2^σ^2^/[*d*^2^(*N* − 1) + *Z*^2^σ^2^], where *N*=58 [the total number of eligible female participants aged 20-50 years in the recruitment setting], *Z*=1.96 [95% confidence level], σ=0.4 [estimated SD of the mean [25]], and *d*=0.05 [5% margin of error]), yielding an n value of 47.08, rounded up to 48. As all 55 eligible volunteers enrolled, the achieved sample exceeded this minimum.

Participants had a mean age of 24.13 (SD 7.99; range 20‐50) years and came from diverse educational backgrounds. Eligibility criteria included smartphone ownership, ability to use mobile apps, and self-reported no prior formal training in BSE. Participants used the VCheck app for a 2-month period before completing the evaluation questionnaires.

### Study Procedure

#### Overview

**Figure 1.**
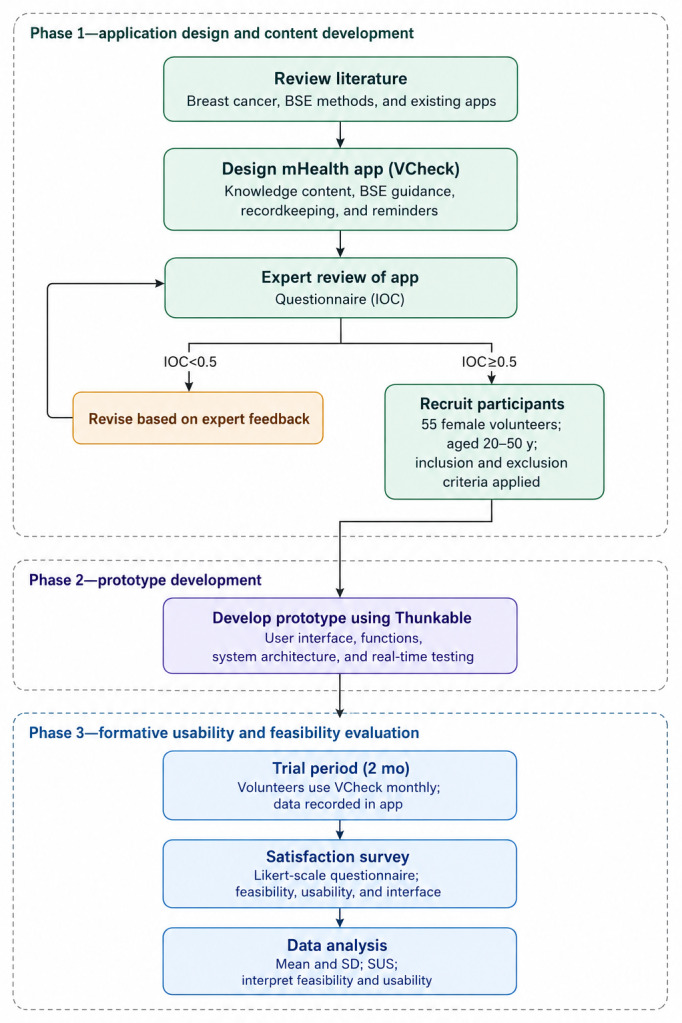
Study workflow for the development and formative evaluation of the VCheck mobile health (mHealth) app. The workflow consisted of 3 phases: phase 1 involved app design and content development, including literature review, content selection, and expert validation using the index of item-objective congruence (IOC); phase 2 involved prototype development using the Thunkable cross-platform environment with block-based visual programming and Firebase integration; and phase 3 involved formative usability and feasibility evaluation. Items with IOC values below 0.5 were revised and re-evaluated prior to phase 3. Phase 3 was conducted among 55 female volunteers aged 20 to 50 years in Thailand over a 2-month trial period. Data were collected via an online questionnaire and analyzed using the System Usability Scale (SUS) and descriptive statistics. BSE: breast self-examination.

This study was conducted in 3 sequential phases (Figure 1).

#### Phase 1: App Design and Content Development

The VCheck app was designed through a structured user-centered process consisting of literature review, content selection, prototype development, expert validation, and pilot usability testing [26]. Educational content and module functions were selected to address common barriers to regular BSE practice, including limited knowledge, uncertainty regarding the correct technique, lack of visual guidance, and absence of reminder support. Content was informed by published literature on breast cancer awareness and BSE, national breast health recommendations, and prior mHealth studies. The content and questionnaire items were reviewed by 3 experts in medical informatics and health promotion using the index of item-objective congruence (IOC); items with IOC values below 0.5 were revised before pilot-testing. On the basis of this structured design process, 4 app modules were developed. The purpose, evidence base, and expected user benefit of each module are summarized in Table 1.

**Table 1.** Design rationale, evidence base, and expected user benefits of the 4 functional modules developed during phase 1 of the evaluation of the VCheck mobile health (mHealth) app for breast self-examination (BSE) among Thai women.

	Purpose	Evidence base	Expected user benefit
Module 1: breast cancer knowledge	Improve breast health awareness and cancer literacy	Breast cancer education literature and Thai national cancer awareness guidelines	Increased breast health knowledge and awareness of changes requiring clinical evaluation
Module 2: step-by-step BSE instructions	Standardize BSE technique and address procedural uncertainty	Thai Department of Health BSE recommendations and evidence-based BSE technique guidelines	Improved confidence and accuracy in performing BSE
Module 3: animated BSE demonstration	Provide visual-procedural guidance to support comprehension of correct BSE technique	mHealth learning theory, multimedia learning principles, and prior animated health education studies	Better comprehension and retention of BSE procedure and reduced technique uncertainty
Module 4: monthly BSE reminder system	Support behavioral reinforcement and improve adherence to monthly BSE practice	Health belief model (cues to action), reminder-based mHealth intervention studies, and behavioral self-regulation theory	Improved regularity and adherence to monthly BSE through timely behavioral cues

#### Phase 2: Prototype Development and System Architecture

The VCheck prototype was developed using the Thunkable cross-platform environment (Thunkable, Inc) following a UCD approach to ensure alignment with user needs and cultural context. Interface components, including text, images, videos, and interactive buttons, were designed within the Thunkable workspace, and app logic was implemented using the Blocks visual programming module. User data management was supported through Firebase (Google) integration, and cloud-based data storage was facilitated using Thunkable data sheets linked with Google Apps Script to enable automated monthly reminder notifications.

**Figure 2.**
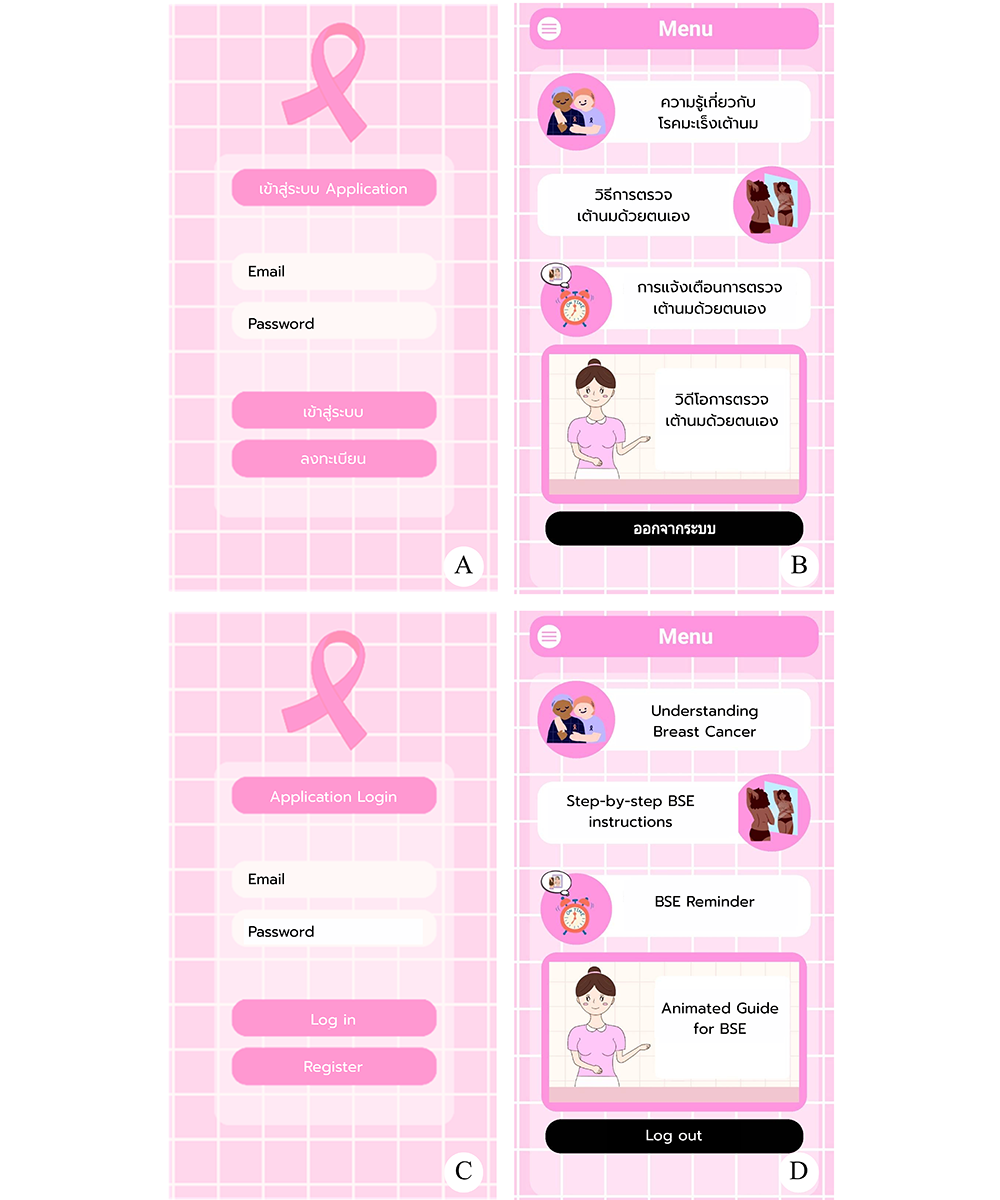
Representative screenshots of the VCheck mobile health app evaluated during a formative usability and feasibility study among 55 Thai women aged 20 to 50 years in Bangkok, Thailand (2023). Panels A and C show the Thai- and English-language log-in interfaces, respectively. Panels B and D show the Thai- and English-language main menus presenting the 4 functional modules: breast cancer education, step-by-step breast self-examination (BSE) instructions, an animated BSE demonstration, and a monthly BSE reminder. The prototype evaluated by participants was presented in Thai; the English-language interfaces are translations provided for international readers.

Real-time testing was conducted using Thunkable Live to verify app functionality and system stability. The prototype was distributed to participants via a web preview link generated through the Thunkable Live environment, which allowed for testing across devices without requiring a native iOS or Android installation. As a result, the current prototype was not compiled as a stand-alone native app for either platform; the participants’ recommendation to develop a fully functional version compatible with both Android and iOS therefore reflects the intended transition from web-based prototype testing to native app deployment in future development phases. The final app comprised four functional modules: (1) breast cancer knowledge, (2) step-by-step BSE instructions, (3) animated demonstrations of BSE techniques, and (4) a reminder system designed to support monthly BSE practice (Figure 2).

#### Phase 3: Formative Usability and Feasibility Evaluation

A structured 10-item questionnaire was developed to assess app feasibility (5 items) and interface usability (5 items) using a 5-point Likert scale. Content validity was evaluated using the IOC method, and items with IOC values below 0.5 were revised or removed based on expert feedback. The finalized questionnaire was distributed electronically via Google Forms (Multimedia Appendix 1).

In addition to the Likert-scale items, each questionnaire item included an open-ended field inviting participants to record suggestions or comments related to that aspect of the app. All 55 participants completed the open-ended fields. Qualitative feedback was analyzed using content analysis: responses were read in full, inductively grouped into thematic categories by the research team, and reported as key themes with representative examples.

Participants attended an orientation session before independently using the app for 2 months (October 17, 2023-December 17, 2023). During the evaluation period, reminder notifications were activated. After the study period, participants completed the satisfaction questionnaire and the System Usability Scale (SUS). The internal consistency of the questionnaire demonstrated satisfactory reliability (Cronbach α>0.8).

### Usability Assessment

Usability was assessed using the Thai version of the SUS, a validated instrument widely used in Thai health research. The SUS consists of 10 items scored on a 5-point Likert scale and yields a composite score ranging from 0 to 100. Scores of 68 or higher indicate above-average usability, and scores of 80 or higher indicate excellent usability.

### Data Privacy and Security

User authentication was implemented through Firebase. The prototype collected only the minimum information required for app use, including email address for account registration, reminder-related information such as menstrual cycle date, and user-entered BSE record data. No medical images, clinical diagnoses, or hospital medical records were collected through the app. App data were stored using cloud-based services connected through Thunkable data sheets and Google Apps Script. Access to the development database was restricted to the research team members responsible for app testing and data management. Data used for analysis were exported in deidentified form. Data transmission relied on secure web-based connections provided by the third-party platforms. As this was a formative prototype, the system was not intended for clinical deployment and was not certified as a medical device or hospital-grade health information system. Future versions will require formal security testing, privacy-by-design review, and compliance assessment before wider implementation.

### Data Analysis

Questionnaire data were analyzed using descriptive statistics. Means, SDs, minimum values, and maximum values were calculated to summarize participant responses. Satisfaction was categorized into 5 levels (very high, high, moderate, low, and very low) using predefined score intervals to facilitate interpretation of Likert-scale responses (Table 2). These categories were defined by the study team for descriptive purposes.

Analyses were conducted at three levels: (1) item-level evaluation, (2) domain-level assessment of feasibility and interface design, and (3) overall satisfaction and usability evaluation using aggregated questionnaire scores and SUS results. SUS scores were calculated according to standard scoring procedures and interpreted using established usability benchmarks.

All analyses were exploratory and intended to inform the formative evaluation of the VCheck app rather than test hypotheses.

**Table 2.** Classification criteria for user satisfaction levels (Likert scale; 1‐5) applied in phase 3 of the VCheck formative evaluation involving 55 female volunteers aged 20 to 50 years (Thailand, 2023) in 2 domains: feasibility and interface design.

Score range	Satisfaction level
4.21‐5.00	Very high
3.41‐4.20	High
2.61‐3.40	Moderate
1.81‐2.60	Low
1.00‐1.80	Very low

### Ethical Considerations

This study was approved by the Human Research Ethics Committee of Vajira Hospital, Navamindradhiraj University (COA 173/2566). Written informed consent was obtained from all participants.

## Results

### Item-Level Analysis of Satisfaction Questionnaire

#### Feasibility of Real-World Implementation

Participants reported high mean satisfaction with the feasibility of the VCheck app for real-world use, with item means ranging from 4.25 (SD 0.69) to 4.62 (SD 0.49). Perceived usefulness of the app for BSE and its role in supporting BSE practice received the highest mean scores (4.62, SD 0.49 and 4.62, SD 0.59, respectively). Intention to use the app in real-life settings was given a mean score of 4.36 (SD 0.67), whereas perceived necessity of the app achieved a mean score of 4.25 (SD 0.69). Agreement regarding the feasibility of real-world implementation achieved a mean score of 4.55 (SD 0.63), indicating strong user acceptance and perceived practicality.

The internal consistency of the feasibility domain was high (Cronbach α=0.89), indicating good reliability of the questionnaire items. SUS scores indicated excellent overall usability performance, suggesting that users perceived the app as highly usable and feasible (Table 3).

**Table 3.** Mean scores, SDs, maximum and minimum scores, observed response levels (ie, the total count of distinct options respondents chose), and score intervals for each questionnaire item: data from phase 3 of the VCheck formative evaluation involving 55 female volunteers aged 20 to 50 years (Bangkok, Thailand, 2023)^a^.

Item	Score, mean (SD)	Min-max	Observed response levels, n	Score interval
Feasibility of real-world app use				
The app is useful for breast self-examination.	4.62 (0.49)	4-5	2	0.50
I would like to use this app in real-life settings.	4.36 (0.67)	3-5	3	0.67
The app is necessary for breast self-examination.	4.25 (0.69)	2-5	4	0.75
The app helps support breast self-examination.	4.62 (0.59)	3-5	3	0.67
I am satisfied with the feasibility of using this app.	4.55 (0.63)	3-5	3	0.67
App interface design				
The app is easy to use.	4.49 (0.60)	3-5	3	0.67
The content and images are appropriate and easy to understand.	4.62 (0.59)	3-5	3	0.67
The app design is visually appealing.	4.36 (0.91)	1-5	5	0.80
The app components are appropriate.	4.36 (0.72)	2-5	4	0.75
The app functions meet user needs.	4.35 (0.69)	3-5	3	0.67

a Items assessed feasibility and interface design using a 5-point Likert scale.

#### App Interface Design

Participants also reported high satisfaction with the app’s interface design and usability (Multimedia Appendix 2). Ease of use achieved a mean score of 4.49 (SD 0.60), whereas clarity and appropriateness of educational content and images received the highest mean score in this domain (4.62, SD 0.59). Visual appeal and appropriateness of layout and interface components were both rated with mean scores of 4.36 (SD 0.91 and 0.72, respectively). Functionality meeting user needs received a mean score of 4.35 (SD 0.69), indicating consistently positive perceptions of usability and interface quality. Internal consistency for the interface design domain was satisfactory (Cronbach α=0.87).

### Analysis of Each Domain

Satisfaction was further analyzed across two domains: (1) feasibility of real-world implementation and (2) app interface format. As shown in Table 4, the feasibility domain achieved a mean satisfaction score of 4.48 (SD 0.64), whereas the interface format domain received a mean score of 4.44 (SD 0.72), both categorized as very high satisfaction levels.

The close alignment between domain scores suggests that both functional feasibility and interface usability contributed comparably to overall satisfaction. According to the SUS interpretation framework, these results correspond to an excellent usability level. The overall mean SUS score was 85.4 (SD 6.8), indicating excellent usability.

**Table 4.** Mean scores, SDs, maximum and minimum scores, observed response levels, and score intervals for each of the 2 evaluation domains assessed in phase 3 of the VCheck formative study involving 55 female volunteers aged 20 to 50 years (Bangkok, Thailand, 2023).

Item	Score (1-5), mean (SD)	Min-max	Observed response levels, n	Score interval
Feasibility of real-world app use	4.48 (0.64)	2-5	4	0.75
App interface design	4.44 (0.72)	1-5	5	0.80

### Overall Satisfaction With the App Trial

Overall satisfaction was evaluated by aggregating scores across both domains. The mean overall satisfaction score was 4.46 (SD 0.68), with a maximum score of 5 and a minimum score of 1. On the basis of the 5-point Likert scale, the calculated score interval was 0.8, indicating adequate differentiation across response categories.

According to the predefined satisfaction criteria, the overall score corresponded to a very high satisfaction level. These findings suggest that participants reported very high satisfaction with both the feasibility and interface design of the VCheck app, indicating strong user acceptance and favorable perceptions of its potential for real-world use.

### User Suggestions for the BSE App

All 55 participants provided suggestions via the questionnaire. Responses were analyzed using content analysis and grouped into thematic categories. Common suggestions included developing a fully functional version compatible with both Android and iOS platforms. To improve accessibility, participants recommended adjustable font sizes, multilingual interface options, and reducing text volume supplemented with clearer illustrative images of breast abnormalities.

Participants also suggested improving system stability and performance to ensure reliable operation during use. These recommendations identify key areas for refinement to support broader adoption and inform future iterations of the app.

## Discussion

### Principal Findings

This formative pilot evaluation provides preliminary evidence that the VCheck app, developed using a culturally adapted UCD approach, achieved high usability (mean SUS score 85.4, SD 6.8) and very high user satisfaction (mean 4.46, SD 0.68) among Thai women. These findings suggest that culturally tailored mHealth tools may support engagement in preventive breast health practices, particularly in settings with limited access to formal screening services.

Usability in this study was operationalized in accordance with the International Organization for Standardization (ISO) 9241-11 framework, which defines usability as the extent to which a product can be used by specific users to achieve specified goals with effectiveness, efficiency, and satisfaction in a specified context of use [27]. Within this framework, the mean SUS score of 85.4 reflects the satisfaction dimension of usability, indicating that participants found VCheck subjectively easy to use and acceptable for supporting self-care BSE practice. The high feasibility ratings further reflect the effectiveness dimension, suggesting that users perceived the app as capable of supporting their intended health behavior. Although objective measures of efficiency (eg, task completion time and error rate) were not assessed in this formative prototype phase, the convergence of high quantitative satisfaction scores and positive qualitative feedback provides initial evidence that VCheck satisfies the core dimensions of usability as defined by the ISO 9241-11 framework. Future studies should incorporate objective usability metrics to more comprehensively evaluate all 3 dimensions of the framework.

The high feasibility rating (mean 4.48, SD 0.64) indicates that participants perceived VCheck as a practical and supportive tool for promoting BSE. This aligns with the intended formative purpose of the app as an educational and behavioral support tool rather than a diagnostic intervention.

### Comparison With Prior Work

These findings are consistent with those of previous mHealth studies demonstrating increased BSE awareness, confidence, and engagement following mobile-based interventions. Prior studies by Yusuf et al [17] and Heo et al [28] have reported improved perceived benefits and engagement among users of smartphone-assisted BSE apps. Similarly, participants in the present study recognized VCheck as a useful tool for supporting self-management of breast health.

High satisfaction with the app’s interface design (mean 4.44, SD 0.72) further supports its usability. Participants highlighted ease of use, visual appeal, and clarity of instructional content and imagery. These outcomes are consistent with those of earlier studies by Kongjit et al [14] and Tiensap et al [29], which reported favorable user responses to mobile apps designed for breast health education and screening awareness. The integration of step-by-step guidance, animated demonstrations, and reminder functions may have contributed to user engagement.

From a human-computer interaction perspective, the observed usability outcomes may be attributed to simplified navigation, consistent interface structure, reduced textual burden, and animation-based demonstrations. These design features are consistent with established usability heuristics related to learnability, efficiency, and user control. In addition, reminder cues and guided visual instructions align with constructs of the health belief model, potentially supporting perceived self-efficacy and motivation to perform regular BSE.

Compared with BSE-related mHealth apps evaluated in other low- and middle-income countries, which often report SUS scores in the mid-60s to mid-70s, the SUS score observed in this study was higher. This suggests that cultural tailoring, Thai-language content, and context-specific visual materials may enhance perceived usability and user experience. This emphasis on context-specific, person-centered design is consistent with the World Health Organization global strategy on digital health [30].

### Limitations

This study has several limitations. First, the evaluation was conducted using a prototype web-based version of the VCheck app rather than a fully developed native mobile app for Android or iOS platforms. System performance characteristics such as responsiveness, loading time, and error handling may differ in real-world deployment, and the findings should be interpreted with caution.

Second, although the Thai-adapted SUS was used to assess perceived usability, other validated multidimensional evaluation instruments such as the Mobile App Rating Scale were not applied. This may limit comparability with other mHealth usability studies and restrict the breadth of usability assessment. These limitations reinforce the need for systematic quality criteria when evaluating mHealth apps [31].

Third, objective usability metrics—including task completion time, navigation efficiency, and error rates—were not measured. Incorporating performance-based indicators aligned with ISO 9241-11 standards could provide a more comprehensive evaluation of system efficiency and user interaction.

Fourth, the sample size was relatively small and consisted primarily of young adult volunteers with relatively high digital literacy, which may limit external validity for older adults or populations with lower technology proficiency.

Finally, long-term behavioral and clinical outcomes—such as sustained app use, adherence to regular BSE, improvement in examination technique, or early detection of breast abnormalities—were not assessed. Therefore, conclusions regarding long-term effectiveness cannot be drawn.

Future studies should include more diverse populations, incorporate objective usability metrics, apply additional validated evaluation tools, and assess longitudinal behavioral outcomes to determine the effectiveness, scalability, and real-world impact of the VCheck app. Despite these limitations, this study provides initial evidence that a culturally tailored Thai-language mHealth app can achieve excellent usability and high user acceptance in a real-world population.

Additionally, digital access and digital literacy represent potential barriers to broader scale-up of mHealth interventions such as VCheck [32]. The current sample consisted primarily of young adult volunteers with relatively high digital literacy and smartphone access. Older women, those in rural areas, or those with lower technology proficiency may face challenges in adopting and sustaining use of the app. Future iterations should consider low-literacy interface design, community-based implementation support, and evaluation in populations with varying levels of digital access to ensure that mHealth tools do not inadvertently widen existing health disparities.

### Conclusions

This formative study developed and evaluated VCheck, a culturally tailored mHealth app designed to support breast health awareness and BSE education among Thai women. The app demonstrated excellent usability and very high user satisfaction. These findings suggest that VCheck is acceptable and usable as a prototype self-care support tool for breast health awareness and BSE education. The present study did not evaluate clinical outcomes, screening performance, or breast cancer detection.

Further studies should evaluate the app’s effectiveness in improving BSE knowledge, technique, adherence, and longer-term implementation outcomes in more diverse populations. Future iterations will incorporate user feedback to improve functionality, accessibility, and alignment with formal health data security standards.

## Supplementary material

10.2196/92230 Multimedia Appendix 1 User satisfaction and usability questionnaire developed to assess the feasibility, usability, and interface design of the VCheck breast self-examination smartphone app. Responses are provided on a 5-point Likert scale.

10.2196/92230 Multimedia Appendix 2 Representative screenshots of the VCheck smartphone app. The app includes a home page, log-in interface, main menu, breast self-examination (BSE) procedure module, examination result recording function, and an automated BSE reminder system. The original app was developed in Thai; English translations are provided in this appendix to facilitate understanding for international reviewers and readers.

## Abbreviations

BSE: breast self-examination
CBE: clinical breast examination
IOC: index of item-objective congruence
ISO: International Organization for Standardization
mHealth: mobile health
SUS: System Usability Scale
UCD: user-centered design
