# Supplementary material for: A Culturally Tailored mHealth App (VCheck) to Support Breast Self-Examination Among Thai Women: Development and Formative Evaluation Study
Source: JMIR Form Res. 2026 Aug 12;10:e92230. doi: 10.2196/92230 (PMC13465625; doi:10.2196/92230)
Supplement: Supplementary file 1 [file formative-v10-e92230-s001.docx]

**Multimedia Appendix 1. User Satisfaction and Usability Questionnaire**

This questionnaire was developed to assess the feasibility, usability, and interface design of the VCheck breast self-examination smartphone application. Responses are rated on a 5-point Likert scale.

Response Scale

5 = Strongly agree

4 = Agree

3 = Neutral

2 = Disagree

1 = Strongly disagree

| **Item** | **Response Scale** | | | | |
| --- | --- | --- | --- | --- | --- |
|  | **5** | **4** | **3** | **2** | **1** |
| **Feasibility of real-world application use** | | | | | |
| 1. The application is useful for breast self-examination |  |  |  |  |  |
| 2. I would like to use this application in real-life settings |  |  |  |  |  |
| 3. The application is necessary for breast self-examination |  |  |  |  |  |
| 4. The application helps support breast self-examination |  |  |  |  |  |
| 5. I am satisfied with the feasibility of using this application |  |  |  |  |  |
| **Application interface design** | | | | | |
| 1. The application is easy to use |  |  |  |  |  |
| 2. The content and images are appropriate and easy to understand |  |  |  |  |  |
| 3. The application design is visually appealing |  |  |  |  |  |
| 4. The application components are appropriate |  |  |  |  |  |
| 5. The application functions meet user needs |  |  |  |  |  |

**Additional Comments**

Please provide any suggestions for improving the application:

....................................................................................................................................................................................................................................................................................................................................................................................................................................................................................

**Methodological Description for Manuscript**

A researcher-developed satisfaction questionnaire consisting of 10 items was used to evaluate feasibility and usability of the VCheck application. The questionnaire included two domains: feasibility of real-world implementation (5 items) and application interface and usability (5 items). Responses were rated on a 5-point Likert scale. Content validity was assessed using the Index of Item–Objective Congruence (IOC), and internal consistency reliability was evaluated using Cronbach’s alpha.

**Content Validity (IOC Statement)**

Content validity was assessed by three experts in medical informatics and health promotion using the Index of Item–Objective Congruence (IOC). Items with IOC values ≥0.5 were retained, while items with lower scores were revised or removed based on expert feedback.

**Reliability (Cronbach’s Alpha Statement)**

Internal consistency reliability of the questionnaire was evaluated using Cronbach’s alpha. The instrument demonstrated satisfactory reliability (Cronbach’s α > 0.80), indicating high internal consistency.
