# Supplementary material for: A Culturally Tailored mHealth App (VCheck) to Support Breast Self-Examination Among Thai Women: Development and Formative Evaluation Study
Source: JMIR Form Res. 2026 Aug 12;10:e92230. doi: 10.2196/92230 (PMC13465625; doi:10.2196/92230)
Supplement: Supplementary file 2 [file formative-v10-e92230-s002.docx]

**Multimedia Appendix 2:**

Figure 1. Example of the VCheck Application Interface

Representative screenshots of the VCheck smartphone application. The application includes a home page, login interface, main menu, breast self-examination (BSE) procedure module, examination result recording function, and an automated BSE reminder system.

The original application was developed in Thai; English translations were provided in this figure to facilitate understanding for international reviewers and readers.

A	B	C	D

A: Home page and branding interface.

B: Login and registration interface.

C: Main menu with BSE educational module, animated guidance, and logging function.

D: BSE educational module

Figure 2. Example of the VCheck Application Interface

A	B	C	D

A: Breast self-examination (BSE) procedure module providing step-by-step visual instructions and guidance on abnormal signs and symptoms.

B: Examination results interface allowing users to document findings and review post-examination recommendations.

C: Examination record interface displaying longitudinal self-examination history, including dates and results of previous assessments.

D: Automated breast self-examination reminder module that schedules notifications based on menstrual cycle information or monthly intervals.

**Table 1. Description of VCheck Application Modules**

| **Module** | **Description** |
| --- | --- |
| Breast Cancer Knowledge | Educational content explaining breast cancer risk factors and symptoms |
| BSE Procedure | Step-by-step instructional and animated guidance for breast self-examination |
| Examination Results | User input and documentation of BSE findings |
| Examination Record | Longitudinal log of self-examination history |
| BSE Reminder | Automated reminder scheduling based on menstrual cycle or monthly intervals |

**ISO 9241 Usability Framework Description**

Usability was conceptualized according to ISO 9241-11, which defines usability in terms of effectiveness, efficiency, and user satisfaction in a specified context of use. The VCheck application was designed with simplified navigation, visual guidance, and culturally adapted content to optimize learnability, efficiency, and user satisfaction.

**Video clip** <https://drive.google.com/file/d/1aI9Ve8AanIpJbxj25HY87H2UQ7mrJL7k/view?usp=sharing>
